# Constituents of *Huberantha jenkinsii* and Their Biological Activities

**DOI:** 10.3390/molecules25153533

**Published:** 2020-08-02

**Authors:** Htoo Tint San, Tanawat Chaowasku, Wanwimon Mekboonsonglarp, Ratchanee Rodsiri, Boonchoo Sritularak, Hathairat Buraphaka, Waraporn Putalun, Kittisak Likhitwitayawuid

**Affiliations:** 1 Department of Pharmacognosy and Pharmaceutical Botany, Faculty of Pharmaceutical Sciences, Chulalongkorn University, Bangkok 10330, Thailand; htootintsan@mohs.edu.mm (H.T.S.); boonchoo.sr@chula.ac.th (B.S.); 2 Department of Pharmacognosy, University of Pharmacy, Yangon 11031, Myanmar; 3 Department of Biology, Faculty of Science, Chiang Mai University, 239 Huay Kaew Rd., Chiang Mai 50200, Thailand; tanawat.chaowasku@cmu.ac.th; 4 Center of Excellence in Bioresources for Agriculture, Industry, and Medicine, Chiang Mai University, Chiang Mai 50200, Thailand; 5 Scientific and Technological Research Equipment Center, Chulalongkorn University, Bangkok 10330, Thailand; wanwimon.m@chula.ac.th; 6 Department of Pharmacology and Toxicology, Faculty of Pharmaceutical Sciences, Chulalongkorn University, Bangkok 10330, Thailand; Ratchanee.R@pharm.chula.ac.th; 7 Natural Products for Ageing and Chronic Diseases Research Unit, Faculty of Pharmaceutical Sciences, Chulalongkorn University, Bangkok 10330, Thailand; 8 Faculty of Pharmaceutical Sciences, Khon Kaen University, Khon Kaen 40002, Thailand; hathairat_s@kkumail.com (H.B.); waraporn@kku.ac.th (W.P.)

**Keywords:** *Huberantha jenkinsii*, glucose uptake, α-glucosidase, Parkinsonism, diabetes

## Abstract

The phytochemical investigation of *Huberantha jenkinsii* resulted in the isolation of two new and five known compounds. The new compounds were characterized as undescribed 8-oxoprotoberberine alkaloids and named huberanthines A and B, whereas the known compounds were identified as allantoin, oxylopinine, *N*-*trans*-feruloyl tyramine, *N-trans-p-*coumaroyl tyramine, and mangiferin. The structure determination was accomplished by spectroscopic methods. To evaluate therapeutic potential in diabetes and Parkinson’s disease, the isolates were subjected to assays for their α-glucosidase inhibitory activity, cellular glucose uptake stimulatory activity, and protective activity against neurotoxicity induced by 6-hydroxydopamine (6-OHDA). The results suggested that mangiferin was the most promising lead compound, demonstrating significant activity in all the test systems.

## 1. Introduction

In recent years, non-communicable diseases (NCDs), such as neurodegenerative disorders, cardiovascular diseases, and metabolic syndromes, have become a major global health issue. In several parts of the world, NCDs have caused serious socioeconomic impacts, through increasing impoverishment and slowing down social and economic development [1]. Plants are a natural source of chemopreventive agents for NCDs. A large number of plant-derived products have been shown to possess protective activity against diabetes [2] and neurodegeneration [3]. Currently, our research has been focused on studying plants with potential preventive activity against NCDs, including neurodegenerative disorders and diabetes [4,5,6].

The genus *Huberantha* Chaowasku, a recently established genus in the family Annonaceae, contains 27 species transferred from the genus *Polyalthia* [7,8]. *Huberantha jenkinsii* (Hook. f. and Thomson) Chaowasku is called “Dang nga khao” in Thai or “Taung-Kabut” in Myanmar. It was formerly known as *Guatteria jenkinsii* Hooker and Thomson or *Polyalthia jenkinsii* (Hook. f. and Thomson) Hooker and Thomson [7,8]. The plant has no records of traditional medicinal uses. A recent report on *Polyalthia cinnamomea* describes the isolation of alkaloids with α-glucosidase inhibitory activity [9], suggesting the possible presence of compounds with antidiabetic potential in plants that are in or related to the genus *Polyalthia*. In this study, the chemical components of *Huberamtha jenkinsii* were isolated and evaluated for biological activities related to NCDs, including neuroprotective activity against Parkinson’s disease, α-glucosidase inhibitory potential, and cellular glucose uptake stimulatory properties.

## 2. Results and Discussion

### 2.1. Structural Characterization

**Figure 1:**
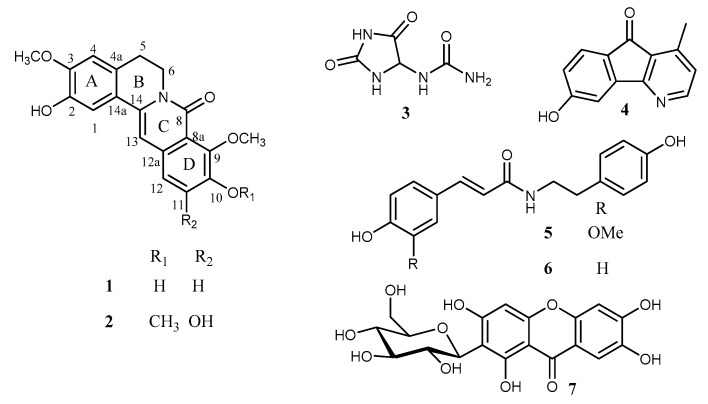
Chemical structures of compounds **1**–**7** isolated from *Huberantha jenkinsii*.

From the stem of *Huberantha jenkinsii*, two new alkaloids (**1** and **2**), together with five known compounds (**3**–**7**), were isolated and structurally characterized (Figure 1). The known compounds were identified as allantoin (**3**) [10], oxylopinine (**4**) [11], *N*-*trans*-feruloyl tyramine (**5**) [12], *N*-*trans*-*p*-coumaroyl tyramine (**6**) [12], and mangiferin (**7**) [13] through the comparison of their spectroscopic data with the literature values.

Compound **1** was obtained as a brownish white powder. The molecular formula was determined to be C_19_H_17_NO_5_ from the deprotonated molecular ion [M-H]^−^ at *m/z* 338.1029 (calcd. for C_19_H_16_NO_5_ 338.1028) in the HR-ESI mass spectrum. The UV spectrum displayed maximal absorptions at 225, 335, and 370 nm, characteristic of the 8-oxoprotoberberine skeleton [14]. The presence of the lactam functionality was supported by the IR band at 1667 cm^−1^ and the ^13^C NMR signal at δ 159.9 (Table 1).

**Figure 2:**
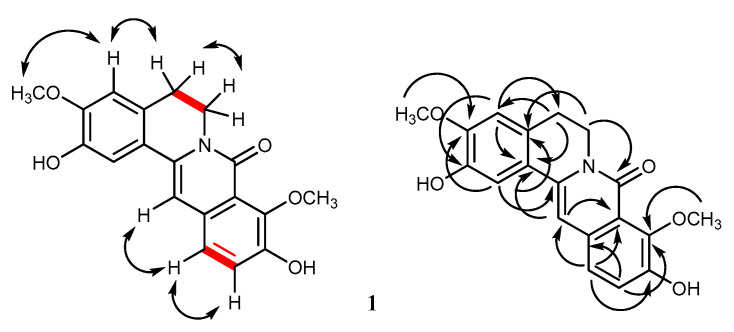
COSY (bold line), NOESY (double arrow line), and HMBC (arrow line) correlations observed for **1**.

The ^1^H NMR spectrum of **1** (Table 1) showed two 2H triplets at δ 2.91 (2H, t, *J* = 6.0 Hz, H_2_-5) and 4.20 (2H, t, *J* = 6.0 Hz, H_2_-6), assignable to the methylene protons of the B ring [15]. This was supported by their correlation peak in the COSY spectrum (Figure 2). The ^13^C NMR resonances for four oxygenated quaternary carbons at δ 146.7, 147.0, 149.4, and 149.5, together with the HSQC correlation peaks for five aromatic methine carbons at δ_H_ 7.33 (1H, s)/δ_C_ 111.8, δ_H_ 7.33 (1H, d, *J* = 8.4 Hz)/δ_C_ 123.7, δ_H_ 7.27 (1H, d, *J* = 8.4 Hz)/δ_C_ 122.6, δ_H_ 6.91 (1H, s)/δ_C_ 101.5, and δ_H_ 6.90 (1H, s)/δ_C_ 111.4, were suggestive of 2,3,9,10-tetraoxygenation [14]. The four oxygen-containing substituents included two phenolic and two methoxy groups, as indicated from the HSQC cross peaks at δ_H_ 3.89 (3H, s)/δ_C_ 56.3 and δ_H_ 3.91 (3H, s)/δ_C_ 62.2. The first methoxy group (δ 3.89) should be placed at C-3, as evident from the NOESY correlation peak between these methoxy protons and H-4, which also showed a NOESY cross peak with H_2_-5 (Figure 2). This proposed structure was corroborated by the HMBC 3-bond correlations from H-4 to the methylene carbon at δ 28.6 (C-5) and the hydroxylated carbon at δ 146.7 (C-2), and from the MeO-3 protons to the carbon at δ 149.4 (C-3) (Table 1 and Figure 2). The second methoxy group (δ 3.91) should be located at C-9 of the D ring, since its protons did not display a NOESY cross peak with H-11. This was supported from the 3-bond connectivity from the MeO-9 protons to C-9 (δ 147.0) and from H-12 to the hydroxylated carbon at δ 149.5 (C-10).

Based on the above spectroscopic properties, **1** was characterized as a new 8-oxoprotoberberine alkaloid with the structure 2,10-dihydroxy-3,9-dimethoxy-5,6-dihydro-8*H*-isoquinolino[3,2-a]isoquinolin-8-one, and given the trivial name huberanthine A. It should be noted that this chemical structure has been mentioned as an in situ intermediate for the organic synthesis of oxypalmatine [16]. However, so far no chemical, physical, or spectroscopic properties of **1** have been described.

**Figure 3:**
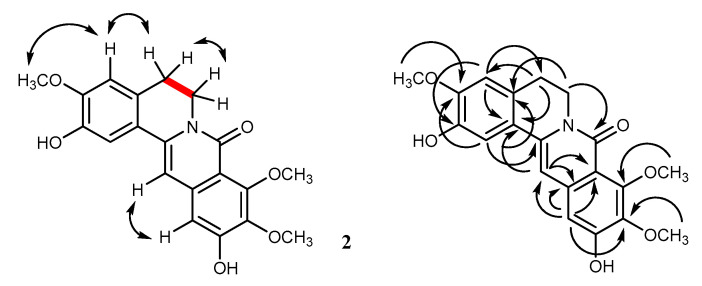
COSY (bold line), NOESY (double arrow line), and HMBC (arrow line) correlations observed for **2**.

Compound **2** had a molecular formula of C_20_H_19_NO_6_, as deduced from the [M-H]^−^ at *m/z* 368.1129 (calcd. for C_20_H_18_NO_6_ 368.1134) in the HR-ESI-MS. The UV absorptions and IR bands of **2** were similar to those of **1**, suggesting an 8-oxoprotoberberine structure. The COSY spectrum showed vicinal coupling for the methylene protons at C-5 and C-6 (Figure 3). It could be inferred from the molecular mass that compound **2** possesses an additional methoxy group, in comparison with **1**. This was supported by the HSQC correlation peaks observed for three methoxy groups at δ_H_ 3.88 (3H, s)/δ_C_ 56.3, δ_H_ 3.90 (3H, s)/δ_C_ 61.5, and δ_H_ 3.90 (3H, s)/δ_C_ 61.9, and for four aromatic methines at δ_H_ 7.33 (1H, s, H-1)/δ_C_ 112.0, δ_H_ 6.90 (1H, s, H-4)/δ_C_ 111.4, δ_H_ 6.85 (1H, s, H-12)/δ_C_ 107.3, and δ_H_ 6.80 (1H, s, H-13)/δ_C_ 100.7 (Table 1). Similar to **1**, compound **2** had a phenolic and a methoxy group located at C-2 and C-3 of ring A, respectively, as evidenced by the NOESY cross peak between the methoxy protons at δ 3.90 and the H-4 proton at δ 6.90 (Figure 3). The two remaining methoxy groups of **2** should be placed at C-9 and C-10, since none of these methoxy protons showed NOESY interaction with H-12 (Figure 3). This was supported by the HMBC correlations from H-12 to C-10 (δ 141.5) and C-11 (δ 155.2) (Figure 3). Thus, **2** was determined to be a new compound, having the structure 2,11-dihydroxy-3,9,10-trimethoxy-5,6-dihydro-8*H*-isoquinolino[3,2-a]isoquinolin-8-one and given the name huberanthine B.

### 2.2. Biological Activities

**Figure 4:**
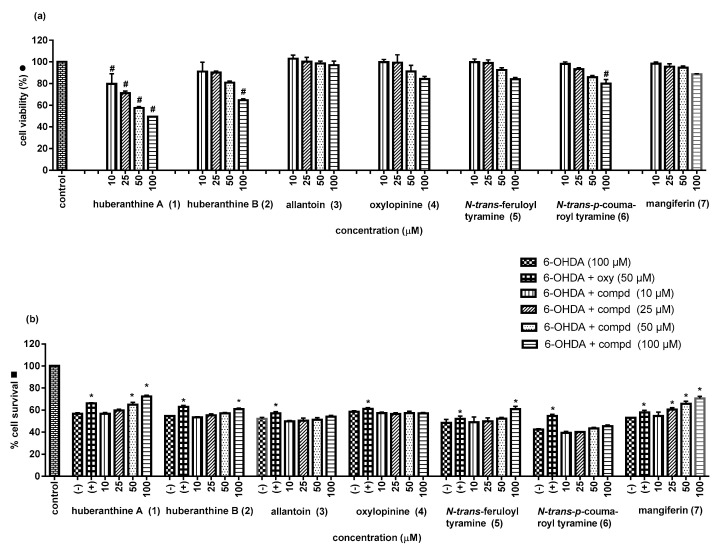
(**a**) Cell viability (● = 24 h incubation with test sample without 6-hydroxydopamine (6-OHDA)) and (**b**) cell survival (■ = 2 h incubation with 6-OHDA after 1 h of pretreatment with test sample); * A *p* < 0.05 is considered to be significantly different from the negative control (6-OHDA 100 µM); # less than 80% cell viability is considered cytotoxic; (−) = negative control (6-OHDA 100 µM); (+) = positive control (6-OHDA 100 µM and oxyresveratrol 50 µM).

The isolated compounds (**1**–**7**) were subjected to assays for protective activity against 6-hydroxydopamine (6-OHDA) in SH-SY5Y cells, an *in vitro* model for preliminary evaluation of neuroprotective potential for Parkinson’s disease (PD) [17,18]. In this study, the neurotoxic agent 6-OHDA (100 µM) was used to cause cell death in the range of 42–58%, whereas oxyresveratrol (50 µM) was employed as the positive control [17,19]. Each of the test compounds was evaluated in four concentrations-i.e., 10, 25, 50 and 100 µM. The percentages of cell viability after a 2 h treatment with 6-OHDA in the presence and the absence of the test compound were obtained and analyzed to determine the percent of cell survival. Cytotoxicity studies of each compound were also conducted by determining the percent cell viability after 24 h of exposure. From Table 2 and Figure 4, it can be seen that the new compounds huberanthines A and B (**1** and **2**), after 2 h incubation, exhibited a moderate activity, with the percent of cell survival of 72.4 ± 1.1% and 60.9 ± 0.9% at 100 µM, respectively. However, after 24 h of exposure, they showed toxicity against the neuronal cells (<80% cell viability as compared to the control).

*N*-*trans*-Feruloyl tyramine (**5**) showed a recognizable neuroprotective potential at 100 μM with a 60.9 ± 2.5% cell survival (6-OHDA: 48.4 ± 3.3% at 100 µM). Compounds **3**, **4**, and **6** were devoid of activity. Interestingly, mangiferin (**7**) displayed a strong neuroprotective activity, with a 60.5 ± 1.7% cell survival at 25 μM (6-OHDA: 53.1 ± 0.5% at 100 μM). In addition, the compound did not show toxicity at all the tested concentrations. The findings in this study were in line with earlier reports on the protective effects of mangiferin against PD [20].

The isolated compounds were further studied for antidiabetic potential. Compounds **3**–**7** were evaluated for α-glucosidase inhibitory activity using established protocols [21,22]. The alkaloids **1** and **2**, however, were not tested due to their limited amounts. Allantoin (**3**) and oxylopinine (**4**) were considered inactive, exhibiting a less than 50% inhibition at 100 µg/mL. The amides *N*-*trans*-feruloyl tyramine (**5**) and *N*-*trans-p*-coumaroyl tyramine (**6**) displayed a strong inhibition of the enzyme, showing IC_50_ values of 30.6 ± 2.9 µM and 0.6 ± 0.1 µM, respectively. Mangiferin (**7**) showed a moderate activity with an IC_50_ value of 253.6 ± 14.2 µM, when compared with the drug acarbose (IC_50_ 724.7 ± 46 µM). The α-glucosidase inhibitory activities of **5**–**7** observed in this study were in agreement with previous reports [23,24].

**Figure 5:**
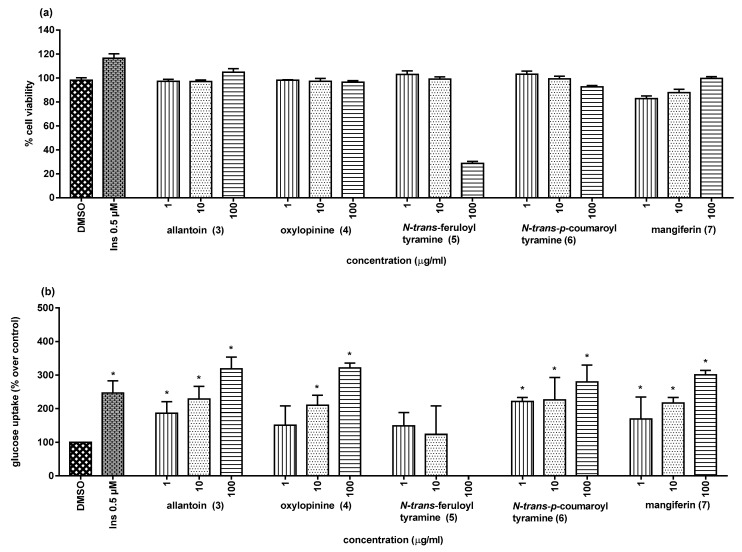
Cytotoxicity (**a**) and glucose uptake stimulation (**b**) of compounds from *H. jenkinsii*. * (*p* < 0.05) Significantly different when compared to the control (DMSO); Ins = insulin (positive control).

Recently, we have found that some α-glucosidase inhibitors (AGIs) of natural origin also possessed the ability to stimulate glucose uptake by skeletal muscle cells [21,22]. This secondary biological activity has currently attracted research attention because it might help to increase the antidiabetic potential of these AGIs. With this in mind, we tested compounds **3**–**7** for their ability to stimulate glucose uptake in L6 myotube cells, with insulin as the positive control. As illustrated in Figure 5, almost all of the tested compounds showed no cytotoxicity against the rat L6 cells at the tested concentrations, except for *N*-*trans*-feruloyl tyramine (**5**), which was toxic when tested at 100 µg/mL (<80% cell viability compared to the control). Under the present experimental conditions, compounds **3**, **4**, **6**, and **7** showed observable cellular glucose uptake stimulatory activity (Table 3 and Figure 5) in comparison with insulin (146.6% at 0.5 µM).

Allantoin (**3**) and mangiferin (**7**) have been earlier reported for cellular glucose uptake enhancing activity [25,26], but oxylopinine (**4**) and *N*-*trans*-coumaroyl tyramine (**6**) have been studied for the first time in this investigation.

It should be noted that, among the compounds evaluated for neuroprotective and antidiabetic activities in this study, mangiferin (**7**) seems to be the most promising, since it showed significant effects in all the three bioassay systems. In fact, this compound has been reported to possess a wide range of pharmacological activities [27]. It has been hypothesized that mangiferin exerts its multiple biological activities, including neuroprotective effects, through its antioxidant property [20]. In a recent study, mangiferin showed synergistic antidiabetic effects with the oral hypoglycemic drugs metformin and gliclazide [28]. Since mangiferin can be obtained in large amounts from the mango tree, *Mangifera indica* L., and several other plants [29], the compound appears to be a potential candidate as a preventive agent for NCDs.

## 3. Materials and Methods

### 3.1. General Experimental Procedures

UV spectra were measured on a Milton Roy Spectronic 300 Array spectrophotometer, and the IR was recorded on a Perkin-Elmer FT-IR 1760x spectrophotometer (Boston, MA, USA). High-resolution electrospray ionization mass spectra (HR-ESI-MS) were recorded with a Bruker micro TOF mass spectrometer (Billerica, MA, USA). NMR spectra were obtained with a Bruker Avance DPX-300 or Avance III HD 500FT-NMR spectrometer (Billerica, MA, USA). MeOH, EtOAc, *n*-butanol, hexane, CH_2_Cl_2_, yeast α-glucosidase, *p*-NPG, glucose oxidase (GO) assay kit, sodium dodecyl sulfate (SDS), 3-(4,5- dimethyl thiazol-2-yl)-5-diphenyl tetrazolium bromide (MTT), and acarbose were obtained from Sigma-Aldrich (St. Louis, MO, USA). Alpha minimal essentialmedium (α-MEM), fetal bovine serum (FBS), and penicillin-streptomycin (10,000 IU/mL) were purchased from Thermo Fisher Scientific (Grand Island, NY, USA). Insulin (100 IU/mL) was acquired from Biocon (Bangalore, India). Silica gel (SiO_2_) and TLC plates were obtained from Merck (Darmstadt, Germany), and Sephadex LH-20 was from Pharmacia (Piscataway, NJ, USA).

### 3.2. Plant Materials

Samples of *Huberantha jenkinsii* were collected from Surat Thani province in April, 2014, and identified by one of us (T.C.). The herbarium specimens have been on deposit at the Faculty of Science, Chiang Mai University.

### 3.3. Extraction, Isolation, and Purification

The stem and leaves showed similar TLC profiles, but the leaves contained large amounts of chlorophylls. The dried stem of (1.1 kg) was chopped and extracted with MeOH at room temperature to give a methanol extract of 180 g after the removal of the solvent. This MeOH extract was treated with EtOAc, *n*-butanol, and water to give corresponding extracts. The EtOAc extract was fractionated by vacuum-liquid chromatography on silica gel (hexane-CH_2_Cl_2_ and CH_2_Cl_2_-MeOH, gradient) to give six fractions (A-F). Fraction B was further separated on Sephadex LH-20 (MeOH) to obtain five fractions (BI-BV). The separation of fraction BII by repeated column chromatography (CC), including reverse-phase CC (C18, MeOH-H_2_O, gradient) and normal phase CC (silica gel; hexane-acetone, gradient), gave compound **1** (3.5 mg), compound **2** (3.4 mg), allatoin (**3**, 32.6 mg), and oxylopinine (**4**, 6.5 mg). Fraction D was separated on Sephadex LH-20 (MeOH) to give four fractions (DI-DIV). *N-trans*-Feruloyl tyramine (**5**, 28.4 mg) was isolated from fraction DIII through purification on CC (silica gel; hexane-acetone, gradient). Fraction E was separated on Sephadex LH-20 (MeOH) to give *N-trans-p*-coumaroyl tyramine (**6**, 6.1 mg). Mangiferin (**7**, 1 g) was obtained as white precipitates from the BuOH extract after it was left standing overnight. It should be mentioned that alkaloids **1** and **2** were quite unstable, as could be seen from their spots on the SiO_2_ TLC plate turning brown in a few minutes. Their purity should be in the range of 90–95%, as estimated from the appearance of the ^1^H NMR spectra.

Compound **1**: brownish white powder; UV (MeOH): λ_max_ (log ε) 205 (3.74), 225 (3.85), 335 (3.60), 370 (3.36) nm; IR: ν_max_ 3353, 1667, 1540, 1513, 1260, 1030, 1095 cm^−1^; HR-ESI-MS: [M–H]^−^ *m/z* 338.1029 (calcd. for C_19_H_16_NO_5_ 338.1028).

Compound **2**: brownish white powder; UV (MeOH): λ_max_ (log ε) 230 (3.85), 260 (3.79), 335 (3.66), 365 (3.49) nm; IR: ν_max_ 3359, 1658, 1632, 1551, 1510, 1268, 1037, 1091 cm^−1^; HR-ESI-MS: [M–H]^−^ *m/z* 368.1129 (calcd. for C_20_H_18_NO_6_ 368.1134).

### 3.4. Assay for Neuroprotective Activity

Cell Culture: SH-SY5Y cells were cultured in growth medium containing DMEM-F12, supplemented with 10% fetal bovine serum (FBS) and 1% penicillin-streptomycin. The cells were maintained under 5% CO_2_ for 2 h. The medium was replaced with new medium every 2 days and subcultured once the cell confluence was 80–90%. There were no more than 50 cell passages to ensure the cell uniformity and reproducibility. 6-OHDA was dissolved in 0.02% ascorbic acid in the same medium to prevent the degradation of 6-OHDA. The toxic dose of 6-OHDA was determined by preliminary screening at different concentrations after 2 h incubation of SH-SY5Y cells at 37 °C under 5% CO_2_ [15,16].

Assay for protective activity against 6-OHDA: The assay was carried out following established methods [17,19]. Neuroblastoma SH-SY5Y cells (5 × 10^4^ cells/well) were seeded into each well. After 24 h incubation of the cells at 37 °C under 5% CO_2_, the sample was added to each well in four different concentrations (10, 25, 50, 100 μM), and the mixture was preincubated for 1 h. Then, the toxicity inducer 6-OHDA (6-hydroxy dopamine hydrobromide) was added, and the mixture was further incubated for 2 h. Ten microliters of resazurin was added to each well 1 h before the end of the incubation to obtain the final resazurin concentration of 0.01 mg/mL. Finally, the neuroprotective activity of each sample against 6-OHDA was determined by measuring the fluorescence intensity at 530 nm excitation and 590 nm emission wavelengths using a microplate reader. Then, the percentage of cell survival was calculated. Oxyresveratrol was used as a positive control.

Cytotoxicity: The assay for the cytotoxicity of each sample was performed in a manner similar to the above-described procedure but in the absence of 6-OHDA, and the mixture was incubated for a longer period of 24 h (instead of 2 h) after the addition of the test sample. The cytotoxicity at each concentration of the sample was described as the percentage of cell viability.

### 3.5. Assay for α-Glucosidase Activity

The assay was carried out following the method in our previous report [4]. It was based on the ability of the sample to inhibit the hydrolysis of *p*-nitrophenyl-d-glucoside (*p*NPG) by α-glucosidase to release *p*-nitrophenol (PNP), a yellow color agent that can be monitored at 405 nm. Briefly, 10 µL of the sample solution and 40 µL of 0.1 unit/mL α-glucosidase were incubated at 37 °C for 10 min. Then, 50 µL of 2 mM *p*NPG was added, and the mixture was further incubated at 37 °C for 20 min. One hundred microliters of 1 M Na_2_CO_3_ was added, and the progress of the enzyme inhibition was observed by measuring the absorbance at 405 nm. Acarbose was used as a positive control.

### 3.6. Assay for Glucose Uptake Stimulatory Activity

The glucose uptake assay was performed following our earlier described protocol [21,22]. Briefly, rat L6 myoblasts were maintained in α-MEM supplemented with 10% fetal bovine serum (FBS) and 1% penicillin-streptomycin at 37 °C under a 5% CO_2_ atmosphere. For the treatment with test compounds, the cells were plated in a 24-well plate at a density of 2 × 10^4^ cells/well. Once the cell reached a 90% confluence, the media were switched to α-MEM with 2% FBS and 1% penicillin-streptomycin (the differentiation medium). The cells were allowed to differentiate into myotubes for 5–7 days, with the media changed every other day. The assay was initiated by incubating the myotubes at 37 °C under 5% CO_2_ for 24 h with the test compound (1, 10, and 100 µg/mL) or insulin (500 nM). The differentiation medium plus 0.1% DMSO was used as the diluent and the negative control. After the incubation, the medium was collected and analyzed for the glucose level using a glucose oxidase assay kit.

Cytotoxicity: Continuously, after the 24 h treatment for the glucose determination, the medium was added to adjust the volume to 200 µL per well. The cells were then treated with 20 µL of the MTT solution (5 mg/mL) and incubated at 37 °C under 5% CO_2_ for 2 h. To dissolve the formazan crystal, to each well was added 200 µL of solubilization solution (40% DMF, 2% glacial acetic acid, 16%w/v SDS in distilled water), and the mixture was shaken for 20 min. Then, the supernatant was collected and measured for the absorbance at 595 nm. Cytotoxicity was expressed as the percent cell viability.

Statistical analysis: The results of the glucose uptake stimulation and cytotoxicity assays were described as the mean ± standard deviation. An analysis of variance was performed using the GraphPad Prism Version 7.00 for Windows (GraphPad Software, Inc., San Diego, CA, USA). The statistical significance of the difference between the means was evaluated using the uncorrected Fisher’s least significant difference post hoc test. A *P* value <0.05 was considered statistically significant.

## 4. Conclusions

This study is the first report of the chemical and biological studies of *Huberantha jenkinsii*. Two new 8-oxoprotoberberine alkaloids, namely huberanthines A and B (**1** and **2**), and five known compounds (**3**–**7**) were isolated and structurally characterized. The isolated compounds were evaluated for their neuroprotective, α-glucosidase inhibitory, and glucose uptake stimulatory activities. The *C*-glucosidic xanthone mangiferin (**7**) appears to be a potential lead compound for the development of preventive agents for NCDs, since it showed significant activity in all the three test systems.

## Figures and Tables

**Table 1:** NMR data of compounds **1** and **2**.

Position	1 *		2 *	
	δ_H_ (Multiplicity, *J* in Hz)	δ_C_	δ_H_ (Multiplicity, *J* in Hz)	δ_C_
1	7.33 (1H, s)	111.8	7.33 (1H, s)	112.0
2		146.7		146.7
3		149.4		149.6
4	6.90 (1H, s)	111.4	6.90 (1H, s)	111.4
4a		128.1		128.4
5	2.91 (2H, t, 6.0)	28.6	2.89 (2H, dd, 6.5, 6.0)	28.7
6	4.20 (2H, t, 6.0)	40.1	4.16 (2H, dd, 6.5, 6.0)	39.7
8		159.9		159.7
8a		119.5		113.1
9		147.0		155.4
10		149.5		141.5
11	7.27 (1H, d, 8.4)	122.6		155.2
12	7.33 (1H, d, 8.4)	123.7	6.85 (1H, s)	107.3
12a		132.9		136.9
13	6.91 (1H, s)	101.5	6.80 (1H, s)	100.7
14		136.1		137.9
14a		123.6		123.4
MeO-2				
MeO-3	3.89 (3H, s)	56.3	3.90 (3H, s)	61.5
MeO-9	3.91 (3H, s)	62.2	3.90 (3H, s)	61.9
MeO-10			3.88 (3H, s)	56.3

* Recorded in acetone-*d*_6_ at 500 and 125 MHz for ^1^H and ^13^C, respectively.

**Table 2:** Neuroprotective activity of compounds **1**–**7**.

Sample	% Cell Survival	Sample	% Cell Survival
Control	100		
*Huberanthine A* (**1**)		*N*-*trans*-*Feruloyl tyramine* (**5**)	
10 μM	56.7 ± 1.2	10 μM	48.9 ± 4.9
25 μM	59.7 ± 1.1	25 μM	49.8 ± 3.3
50 μM	65.0 ± 2.1 *	50 μM	51.8 ± 1.1
100 μM	72.4 ± 1.1 *	100 μM	60.9 ± 2.5 *
6-OHDA (100 µM)	56.8 ± 0.8	6-OHDA (100 µM)	48.4 ± 3.3
Oxyresveratrol (50 µM)	66.0 ± 0.6 *	Oxyresveratrol (50 µM)	56.1 ± 3.3 *
*Huberanthine B* (**2**)		*N*-*trans*-*p*-*Coumaroyl tytyramine* (**6**)	
10 μM	53.6 ± 0.3	10 μM	39.3 ± 1.4
25 μM	55.3 ± 1.3	25 μM	40.2 ± 0.3
50 μM	57.1 ± 0.8	50 μM	43.5 ± 0.8
100 μM	60.9 ± 0.9 *	100 μM	45.5 ± 1.0
6-OHDA (100 µM)	54.7 ± 0.4	6-OHDA (100 µM)	42.5 ± 0.7
Oxyresveratrol (50 µM)	62.9 ± 1.2 *	Oxyresveratrol (50 µM)	54.7 ± 1.5 *
*Allantoin* (**3**)		*Mangiferin* (**7**)	
10 μM	49.7 ± 1.0	10 μM	54.8 ± 3.5
25 μM	50.5 ± 2.3	25 μM	60.5 ± 1.7 *
50 μM	51.3 ± 1.9	50 μM	65.7 ± 2.5 *
100 μM	54.2 ± 1.0	100 μM	70.7 ± 1.8 *
6-OHDA (100 µM)	52.2 ± 1.2	6-OHDA (100 µM)	53.1 ± 0.5
Oxyresveratrol (50 µM)	57.1 ± 1.6 *	Oxyresveratrol (50 µM)	57.9 ± 1.8 *
*Oxylopinine* (**4**)			
10 μM	45.5 ± 1.0		
25 μM	56.5 ± 1.0		
50 μM	57.2 ± 1.9		
100 μM	57.0 ± 0.8		
6-OHDA (100 µM)	58.4 ± 0.9		
Oxyresveratrol (50 µM)	61.0 ± 1.2 *		

* (*p* < 0.05) Significantly different when compared to 6-OHDA (100 µM).

**Table 3:** Glucose uptake stimulatory activity of compounds **3**–**7.**

Sample	% Glucose Uptake	% Enhancement
DMSO	100	0
Insulin (0.5 µM)	246.6 ± 35.8 *	146.6
Allantoin (**3**)		
1 μg/mL (6.32 µM)	186.5 ± 34.3 *	86.5
10 μg/mL (63.24 µM)	228.4 ± 37.7 *	128.4
100 μg/mL (632.44 µM)	318.2 ± 35.5 *	218.2
Oxylopinine (**4**)		
1 μg/mL (4.73 µM)	150.7 ± 57.1	NA
10 μg/mL (47.34 µM)	210.1 ± 29.6 *	110.1
100 μg/mL (473.44 µM)	321.0 ± 14.6 *	221.0
*N*-*trans*-Feruloyl tyramine (**5**)		
1 μg/mL (3.19 µM)	148.7 ± 39.7	NA
10 μg/mL (31.91 µM)	123.7 ± 84.6	NA
100 μg/mL (319.13 µM)	NC	NA
*N*-*trans*-*p*-Coumaroyl tyramine (**6**)		
1 μg/mL (3.53 µM)	221.0 ± 12.5 *	121.0
10 μg/mL (35.29 µM)	225.7 ± 67.3 *	125.7
100 μg/mL (352.95 µM)	279.7 ± 49.9 *	179.7
Mangiferin (**7**)		
1 μg/mL (2.32 µM)	169.6 ± 14.9 *	69.6
10 μg/mL (23.68 µM)	216.9 ± 16.4 *	116.9
100 μg/mL (236.77 µM)	300.7 ± 12.9 *	200.7

* (*p* < 0.05) Significantly different from the control (DMSO); NA = not applicable; NC = not calculated due to toxicity.
